# Biodegradation of diuron by an endophytic fungus *Neurospora intermedia* DP8-1 isolated from sugarcane and its potential for remediating diuron-contaminated soils

**DOI:** 10.1371/journal.pone.0182556

**Published:** 2017-08-15

**Authors:** Yanhui Wang, Honghong Li, Guojun Feng, Liangwei Du, Dongqiang Zeng

**Affiliations:** 1 Institute of Pesticide and Environmental Toxicology, Guangxi University, Nanning, PR China; 2 Guangxi Key Laboratory of Biology for Crop Diseases and Insect Pests, Plant Protection Research Institute, Guangxi Academy of Agricultural Sciences, Nanning, PR China; 3 Guangxi Key Laboratory of Agric-Environment and Agric-Products Safety, Guangxi University, Nanning, PR China; 4 College of Chemistry and Chemical Engineering, Guangxi University, Nanning, PR China; Universidade de Coimbra, PORTUGAL

## Abstract

A diuron-degrading endophyte DP8-1 was isolated from sugarcane root grown in diuron-treated soil in the present study. The endophyte was identified as *Neurospora intermedia* based on the morphological characteristics and sequence analysis. The fermentation parameters including temperature, pH, inoculation size, carbon source, and initial diuron concentration were also investigated for the optimization of degradation efficiency. The results indicated that strain DP8-1 was capable of degrading up to 99% diuron within 3 days under the optimal degrading condition. The study of degradation spectrum indicated that strain DP8-1 could also degrade and utilize fenuron, monuron, metobromuron, isoproturon, chlorbromuron, linuron, and chlortoluron as substrate for strain growth. On basis of liquid chromatography-mass spectrometry analysis for the products of the degradation of diuron, strain DP8-1 metabolized diuron to produce N-(3,4-dichlorophenyl)-urea and N-(3,4-dichlorophenyl)-N-methylurea through sequential N-dealkylations. In a soil bioaugmentation experiment, the inoculation of strain DP8-1 into diuron-treated soil effectively enhanced the disappearance rate of diuron.

## Introduction

Diuron (3-(3,4-dichlorophenyl)-1,1-dimethylurea), a phenylurea herbicide, is widely used as a broad-spectrum herbicide for pre-emergence weed control in a wide variety of crops, especially sugarcane cultures [1]. Diuron is relatively persistent in soil with the mean half-life of approximately 330 days [2], which contaminates surface and ground waters worldwide by runoff [3, 4] and leaching [5, 6]. Diuron is classified as a Priority Hazardous Substance by the European Commission (Directive 2000/60/CE) and accordingly has been banned in Europe but remains used in numerous regions of the world. The pollutant of its residues in the environment affects not only terrestrial and aquatic biota but also human health. Additionally, diuron is suspected to be a carcinogenic and genotoxic compound for humans and aquatic organisms [7, 8]. Therefore, the dissipation of this compound from the environment is a central issue.

The major route for natural dissipation of diuron from the environment is microbial degradation [9]. The diuron-biodegrading potentials were reported about bacteria such as *Arthrobacter* sp. N2 [10, 11], *Acinetobacter johnsonii* [12], *Streptomycete* strains [13], *Micrococcus* sp. PS-1 [14, 15], and *Bacillus*, *Vagococcus*, *and Burkholderia* spp. [16] isolated from soil samples. Diuron-degrading bacteria identified as *Pseudomonas* sp. and *Stenotrophomonas* sp. were isolated from lotic surface water that has been sensitized to diuron exposure for more than 10 years [17]. The degradation potentials for diuron of five soil fungus *Mortierella* strains were compared [18]. The white-rot fungus *Phanerochaete chrysosporium* was reported for its capacity to degrade diuron in liquid stationary cultures [1]. Sørensen et al. examined the degradation and mineralization of diuron at low concentrations by *Sphingomonas* sp. SRS2 from soil samples in a British agricultural field [19]. So far, the majority of studies on the biodegradation of diuron have focused on microbes isolated from soil, sludge, sediment and water samples in various diuron exposed environments.

At present, fewer studies have reported about the biodegradation of herbicides by using endophytic microbes that reside in the internal tissues of plants without causing apparent negative symptoms of infection. It was demonstrated that endophytes play a key role in host plant adaptation in polluted environment [20]. Herbicide-degrading endophytes could be isolated from plants grown in herbicide-treated areas. It was reported that endophytic rhizobacteria for the degradation of simazine were isolated from the roots of corn plants and the nodules of soybean plants [21]. A study isolated endophytic bacteria *Pseudomonas oryzihabitans* and *Burkholderia gladioliwhich* from soybean grown in soil treated with glyphosate herbicide [22]. Recently, an endophytic quinclorac-degrading bacterium *Bacillus megaterium* Q3 was isolated from the root of tobacco grown in quinclorac-contaminated soil [23]. Up to now, it has not been reported endophytic microorganisms able to degrade diuron.

In the present paper, a diuron-degrading endophytic fungus DP8-1 was isolated from the root of sugarcane grown in a diuron-treated field. The morphological characteristics and sequence analysis of this endophyte was investigated to identify it as *Neurospora intermedia*. The effect factors on the degradation efficiency were also studied to find the optimal degrading condition. The degradation capability of this endophyte to other phenylurea herbicides was also investigated. The biochemical degradation pathway of diuron by this endophyte was proposed based on the analysis of degradation products. The endophyte was tested for the bioremediation of diuron-contaminated soil. To our knowledge, it was the first report on the biodegradation of diuron by endophyte from sugarcane.

## Materials and methods

### Materials

The five standards with above 98% purity of diuron, 3,4-dichloroaniline (3,4-DCA), 1-(3,4-dichloro-phenyl)-3-methylurea3-(4-chlorophenyl) methyl urea (DCPMU), 1-(3,4-dichlorophenyl) urea (DCPU), and 3,4-dichloroacetanilide (3,4-DCAA) were purchased from Dr. Ehrenstorfer GmbH (Augsburg, Germany). Acetonitrile (chromatographic grade) was obtained from Sigma Aldrich (USA). All other reagents used in this study were of analytical grade. The standard *N*. *intermedia* BNCC144684 was obtained from Bena Culture Colletion (BNCC, China). Unless otherwise stated, deionized water was used in all of the experiments.

Sugarcane plants were removed from an agricultural filed in Wuming District of Nanning city, Guangxi, China (23°9′8.2476″N, 108°11′54.9828″E) with a previous history of diuron application. No specific permission was required to sampling in this location. The roots of sugarcane plant were collected and placed in a plastic bag and immediately transported to the laboratory. Then, they were thoroughly washed using running tap water to remove soil and a sonication step was employed to dislodge any soil and organic matter from the surface of sugarcane roots.

The mineral salt medium (MSM) contained (g L^–1^): NH_4_NO_3_ 1.0, K_2_HPO_4_ 1.5, KH_2_PO_4_ 0.5, NaCl 1.0, MgSO_4_·7H_2_O 0.1, and FeSO_4_ 0.025. The amendment MSM was prepared by adding 0.5 g L^–1^ soluble starch. The Luria–Bertani’s (LB) medium was composed of 5 g L^–1^ yeast extract, 10 g L^–1^ tryptone, and 10 g L^–1^ NaCl. The composition of potato dextrose (PD) medium for the purification and enlarged cultivation of the isolated endophyte was as follows (g L^–1^): potato extract 3.0 and dextrose 20.0. Solid medium was prepared by adding 1.5% (W/V) agar into above liquid medium.

### Isolation of diuron-degrading endophytic fungus DP8-1

The cleaned sugarcane roots were surface-sterilized using serial washing in 75% (V/V) ethanol for 2 min, deionized water three times with 1 min each, and 0.1% mercuric chloride for 1 min. Finally, the surface-sterilized sugarcane roots were washed by sterile deionized water three times to clean sterilization agent residues. To check the sterilization process, aliquots of sterile deionized water from the final washing step were spread onto LB agar plate and incubated at 28°C for 5 days. The sugarcane roots were considered clean if no colony was found on agar plate after inoculation.

1 g fresh roots were cut up and ground with 10 mL sterile deionized water in a sterile mortar. 100 μL of the grinding suspension was spread onto MSM agar plate containing 500 mg L^–1^ diuron and then cultured at 28°C for 7 days. Colonies with clear zones were selected as potential diuron-degrading endophytic microbes.

### Identification of the endophytic fungus DP8-1

Colony growth and morphology were examined on PD agar plate, following incubation at 28°C for 7 days. Cell morphology, mycelium, and spore were observed under a light microscope.

The fungal strain DP8-1 was molecularly identified by the analysis of partial sequences of β-tubulin (*Bml*), translational elongation factor1-*α* (*tef-1*), protein kinase C (*pkc*), 28S rDNA, mitogen-activated protein kinase-2 (*mak-2*) and a hypothetical protein-coding gene (NCU02332). For *Bml*, two regions were amplified with separate primer pairs, while for the other gene loci one region was amplified. PCR amplifications were performed using a Analytik Jena DNA thermal cycler (Gradient SL96, Germany) under the following conditions: initial denaturation at 95°C for 5 min, followed by 35 cycles of denaturation at 95°C for 1 min, annealing at 53–58°C for 30 s, and extension at 72°C for 1 min, with a final extension at 72°C for 10 min and final hold at 4°C. The primers, annealing temperatures and references for all target nuclear coli were summarized in Table 1. DNA sequencing was determined by the Shanghai Invitrogen Biological Technology Co., Ltd (China) and aligned with the published sequences in GenBank using BLAST program. Phylogenesis was analyzed using MEGA software.

**Table 1:** The primers, annealing temperatures and references for all target nuclear coli.

Gene loci	Primer	Primer DNA sequence (5'‒3')	Annealing temperatures	References
*Bml*	Bt1a	TTCCCCCGTCTCCACTTCTTCATG	58	[24]
	Bt1b	GACGAGATCGTTCATGTTGAACTC		
	Bt2a	GGTAACCAAATCGGTGCTGCTTTC		
	Bt2b	ACCCTCAGTGTAGTGACCCTTGGC		
*tef-1*	tef-1F	RGACAAGRCTCACATCAACGTSGT	53	[25]
	tef-1R	CCAGTRATCATGTTCTTGATGAART		
*pkc*	PkcF	AAGATCGAGCGCGAAAAGGCTCTGATC		
	pkcR	TATCTCTTSARTGCCTGCTTCAAGAG		
28S	LR0R	ACCCGCTGAACTTAAGC	55	[26]
	LR5	ATCCTGAGGGAAACTTC		[27]
*mak-2*	2393F	GAACTGATGGAGACTGACATGC	58	[28]
	2393R	TTCAACTGCTCCTTGCTCA		
NCU02332	2332F	TGAAGAGGGTATTAAGGARATGA	58	
	2332R	GATGCTGACCTCTCCAAG		

### Optimization of diuron-degrading conditions

Temperature, pH, and inoculation amount were important variables for the degradation of diuron by strain DP8-1. The tested ranges were 20–40°C, 5–9, and 0.1–1 g dry wt L^−1^ for temperature, pH, and inoculum, respectively. Response surface methodology (RSM) based on the Box–Behnken design of experiment was used to optimize these parameters and evaluate their interaction which significantly affected the degradation of diuron by strain DP8-1 [29]. Three independent variables were considered, and the total of 15 experimental runs with three replicates were designed and performed in a random order. The experimental parameters and coded levels of independent variables for diuron biodegradation were showed in Table 2. The MSM solution containing 50 mg L^–1^ diuron inoculated with strain DP8-1 was cultured on a rotary shaker at 150 rpm and withdrawn for detecting diuron residues after 3 days. The obtained data were accurately estimated by quadratic polynomial equation using the response surface regression (RSREG) procedure of the Statistic Analysis System software packages (version 8.0).

$$Y_{i}=b_{0}+\sum b_{i}X_{i}+\sum b_{ij}X_{i}X_{j}+\sum b_{ii}X_{i}{}^{2}$$ (1)

where *Y*_i_ is the predicted response, *X*_i_ and *X*_j_ are variables, *b*_0_ is the constant, *b*_i_ is the linear coefficient, *b*_ij_ is the interaction coefficient, and *b*_ii_ is the quadratic coefficient.

**Table 2:** Box-Behnken experimental design with three independent variables.

Run	A	B	C	Responses (residues of diuron, mg L^‒1^)
1	‒1	‒1	0	20.20
2	1	‒1	0	17.73
3	‒1	1	0	16.52
4	1	1	0	9.87
5	‒1	0	‒1	15.33
6	1	0	‒1	11.38
7	‒1	0	1	13.12
8	1	0	1	11.66
9	0	‒1	‒1	10.33
10	0	1	‒1	9.19
11	0	‒1	1	8.31
12	0	1	1	5.18
13	0	0	0	3.72
14	0	0	0	3.95
15	0	0	0	2.73

A, temperature, ‒1 (20°C), 0 (30°C), 1 (40°C); B, media pH, ‒1 (5), 0 (7), 1 (9); C, biomass amount, ‒1 (0.1 g L^‒1^), 0 (0.55 g L^‒1^), 1 (1 g L^‒1^).

## Effect of alternate carbon source on the degradation of diuron

To study the effect of different carbon sources on diuron degradation by strain DP8-1, 0.5% (W/V) carbon sources including soluble starch, sucrose, yeast extract, and glucose were added into MSM with 50 mg L^–1^ diuron. The inoculated solutions in 250-mL Erlenmeyer flasks (in triplicate) were incubated under the optimal conditions for 3 days. Non-inoculated samples were kept as controls. Sample using diuron as the sole carbon source for culture was also used to compare the efficiency of diuron degradation. Additionally, 0.2 mg L^‒1^ biotin was added into MSM with 50 mg L^–1^ diuron to study its effect on the growth and degradability of strain DP8-1.

## Biodegradation of diuron by strain DP8-1

The strain DP8-1 was cultured in 250-mL Erlenmeyer flasks containing 100 mL PD medium supplemented with 50 mg L^–1^ diuron. The strain was collected by centrifugating at 5,000 rpm for 5 min and washed three times with MSM to remove remaining PD medium. Then, 0.55 g dry wt L^–1^ biomass was seeded into 100 mL amendment MSM fortified with 50 mg L^–1^ diuron and incubated at 150 rpm on a rotary shaker. Controls including abiotic test and degradation test by *N*. *intermedia* BNCC144684 were carried out to evaluate the degradation efficiency of strain DP8-1. At regular time intervals, the degradation culture was withdrawn for the determination of strain growth and diuron degradation. The biomass of strain DP8-1 was measured by dry weight of mycelium (in grams per liter), while the concentration of diuron was detected by high performance liquid chromatography (HPLC) equipped with a UV detector and an XDB C_18_ column (250×4.6 mm, 5 μm) (Agilent 1260, USA). Biodegradation of diuron at different initial concentrations of 10‒400 mg L^−1^ and other phenylurea herbicides with the concentration of 50 mg L^–1^ including fenuron, monuron, metobromuron, isoproturon, chlorbromuron, and linuron were also performed under the optimal conditions.

### Determination of the degradation products of diuron

10 mL degradation liquid was centrifuged at 12,000 rpm for 10 min to remove the proteins. The supernatant was mixed with 20 mL ethyl acetate to extract the degradation products. Then, 5 g NaCl was added to make the mixed solutions separate into two phases. The upper phase was taken, dewatered by Na_2_SO_4_, and concentrated before detection by liquid chromatogram-mass spectrometry (LC-MS) with an electrospray ionization (ESI) source (Agilent, USA) [30, 31].

The ESI-MS condition was as follows: the capillary voltage was set to 3,000 V at gas temperature of 350°C with a drying gas flow of 10 L min^‒1^, and the MS was operated in the positive and negative polarity mode. Full scans were obtained by scanning from 100 to 500 m/z.

### Soil bioremediation experiment

The soil samples (the top 0–10 cm) were collected from a farm in Guangxi University, Nanning, China, where was never applied diuron. The soil samples were ground to powder after air drying and passed through a 2 mm sieve and sterilized on 3 consecutive days [32]. The fresh and sterile soils added with 2.5 mg kg^‒1^ diuron were inoculated with strain DP8-1, while soils without strain DP8-1 were used as control. The biomass of this diuron-degrading strain for each experiment was approximately 1 g per 100 g soil. The soil samples were incubated in the dark at 30°C, and the residue of diuron in the soils was determined at different intervals.

## Results and discussion

### Isolation and identification of the diuron-degrading strain

During the isolation of diuron-degrading strains from sugarcane root, a fungal strain was found to degrade approximately 99% diuron in amendment MSM within 3 days under the optimal conditions. This fungal strain was designated as DP8-1 and chosen for the intensive study.

The colony morphology of strain DP8-1 on PD agar plate was shown in S1a Fig. After cultivation for 1 day, the diameter of colony was more than 80 mm. The newborn hyphae of strain DP8-1 were white, and then turned orange red at the top of aerial hyphae after cultivation for 5 days along with the generation of conidia. The color of the colony was grayish-yellow and the reverse face of the colony was light yellow. The surface of the colony was flat and thin with the submerged mycelia and sparse aerial hyphae. Microscopic examination of this strain illustrated in S1b Fig showed that mycelium composed of hyaline to pale brown hyphae, septate, branched and anastomosing, smooth. The conidiophore in S1c Fig was orange red, spherical or oval with 10–12 μm and the conidia surface showed irregular ornamentation in light microscope. The morphological characteristics of strain DP8-1 matched with those of *N*. *intermedia*.

**Fig 1:**
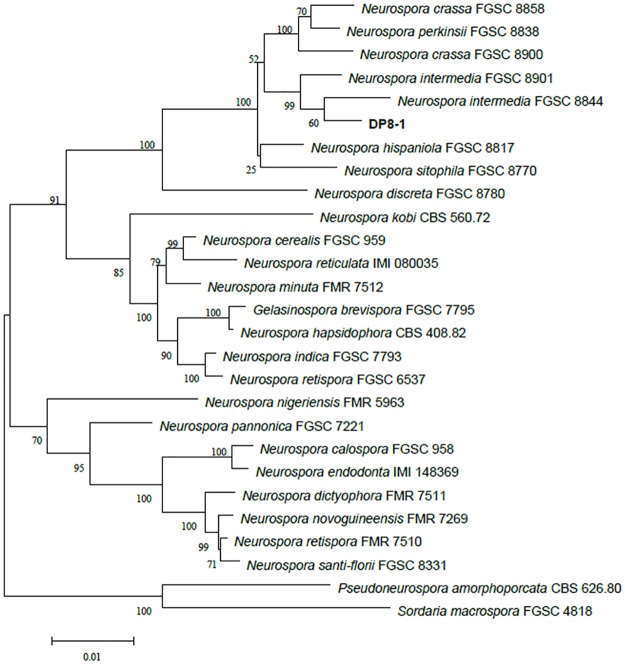
Phylogenetic tree of strain DP8-1 and related by the neighbor-joining approach. Bootstrap values obtained with 1000 repetitions are indicated as percentages at all branches.

Six gene sequences of strain DP8-1 including *Bml*, *tef-1*, *pkc*, 28S rDNA, *mak-2*, and NCU02332 were generated in this study. The concatenated dataset contained a total of 4607 bp. All sequence data of strain DP8-1 acquired for this study had been deposited in the GenBank under the accession numbers MF362950 to MF362955 (www.ncbi.nlm.nih.gov). To analyze the phylogeny of strain DP8-1, strains from 26 taxa including two outgroups were chosen to construct a phylogenetic tree based on concatenated dataset of six gene loci using maximum likelihood bootstrap values of MEGA software and their sequences used in the phylogenetic analysis were downloaded from GenBank. In the phylogeny illustrated in Fig 1, strain DP8-1 clustered together with the heterothallic taxa *N*. *intermedia*, *N*. *crassa*, and *N*. *perkinsii*, forming the branch of the ingroup. This group is monophyletic with full support value, in accordance with earlier report [28]. Phylogenetic analysis of strain DP8-1 showed that strain DP8-1 was closely related to the fungus species of *N*. *intermedia* FGSC 8901 and *N*. *intermedia* FGSC 8844.

According to the results of colonial and microscopic morphologies, and sequence evaluations, strain DP8-1 was identified as *N*. *intermedia*. It was reported that the modern history of Neurospora begins with meterial from sugarcane bagasse and sugarcane appears to be an ideal substrate [33]. Obviously, the sugarcane root could fulfill the requirement of this biotin-auxotrophs fungus. Thus, this endophyte was isolated without added biotin. To our knowledge, this study was the first report that *Neurospora* species degraded diuron.

### Optimization of degradation conditions

The interactive effects of three important variables including temperature, medium pH, and initial inoculation biomass were analyzed using Design-Expert Version 8.0 (Stat- Ease, Inc. Minneapolis, USA). The experimental designs were shown in Table 2. The data were applied multiple regression analysis and the following second-degree polynomial equation was fitted to account for diuron biodegradation by strain DP8-1:

$$Y=3.47-1.82A-1.98B-1.00C-1.04AB+0.62AC-0.50BC+8.62A^{2}+4.00B^{2}+0.79C^{2}$$ (2)

where Y is the biodegradation rate and A, B, and C are independent variables of temperature, pH, and initial inoculation biomass, respectively.

**Fig 2:**
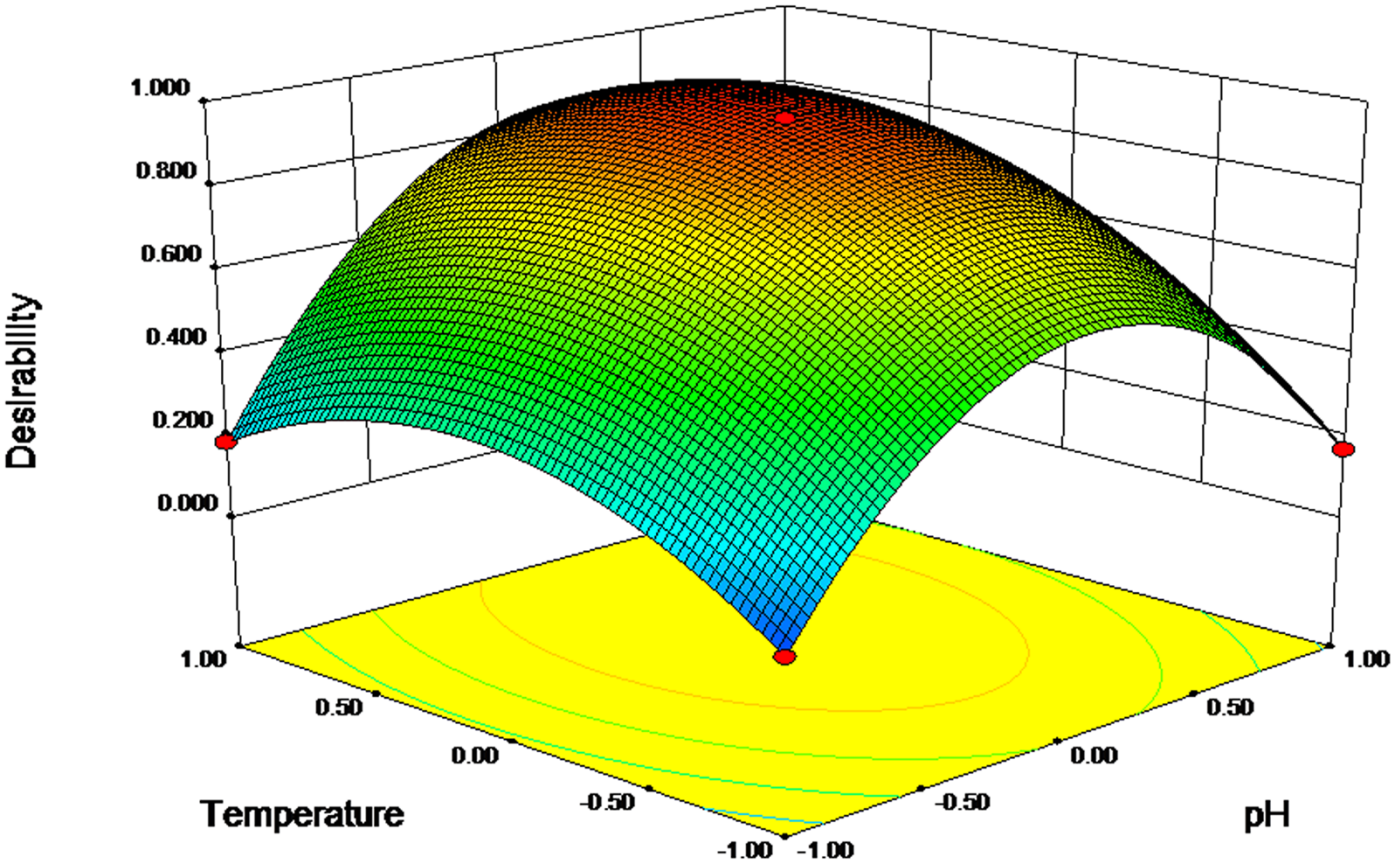
Response surface plot showing the effects of temperature and pH on diuron degradation by strain DP8-1 with inoculum at 0.55 g dry wt L^‒1^.

The results of ANOVA analysis for RSM were shown in Table 3. The accuracy of the model was evaluated using a determination coefficient (R^2^ = 0.9718). The regression model with p value<0.05, F value = 19.13, and a lack of fit value = 0.1098 was statistically acceptable [34, 35]. The results of regression analysis indicated that temperature and pH were significantly affected the degradation rate of diuron by strain DP8-1 (p<0.05), which was consistent with previous report [36]. The three-dimensional response surface plot in Fig 2 displayed the effects of temperature and pH on the biodegradation activity of strain DP8-1 when the inoculation biomass maintained at the fixed value of 0.55 g dry wt L^‒1^. The theoretical maximum response value was obtained when strain DP8-1 was cultured at 32.6°C and pH 7.2.

**Table 3:** ANOVA analysis for the response surface quadratic model.

Source	Sum of squares	df	Mean square	F value	p-value prob>F	
*model*	387.93	9	43.10	19.13	0.0023	significant
*A-pH*	26.39	1	26.39	11.71	0.0188	
*B-Temperature*	31.24	1	31.24	13.86	0.0137	
*C-Biomass*	7.92	1	7.92	3.51	0.1197	
*AB*	4.37	1	4.37	1.94	0.2226	
*AC*	1.55	1	1.55	0.69	0.4447	
*BC*	0.99	1	0.99	0.44	0.5368	
*A2*	274.14	1	274.14	121.64	0.0001	
*B2*	58.98	1	58.98	26.17	0.0037	
*C2*	2.30	1	2.30	1.02	0.3588	
*Residual*	11.27	5	2.25			
*Lack of Fit*	10.43	3	3.48	8.27	0.1098	not significant
*Pure Error*	0.84	2	0.42			
*Cor Total*	399.20	14				

### Effect of carbon source on the degradation of diuron by strain DP8-1

**Fig 3:**
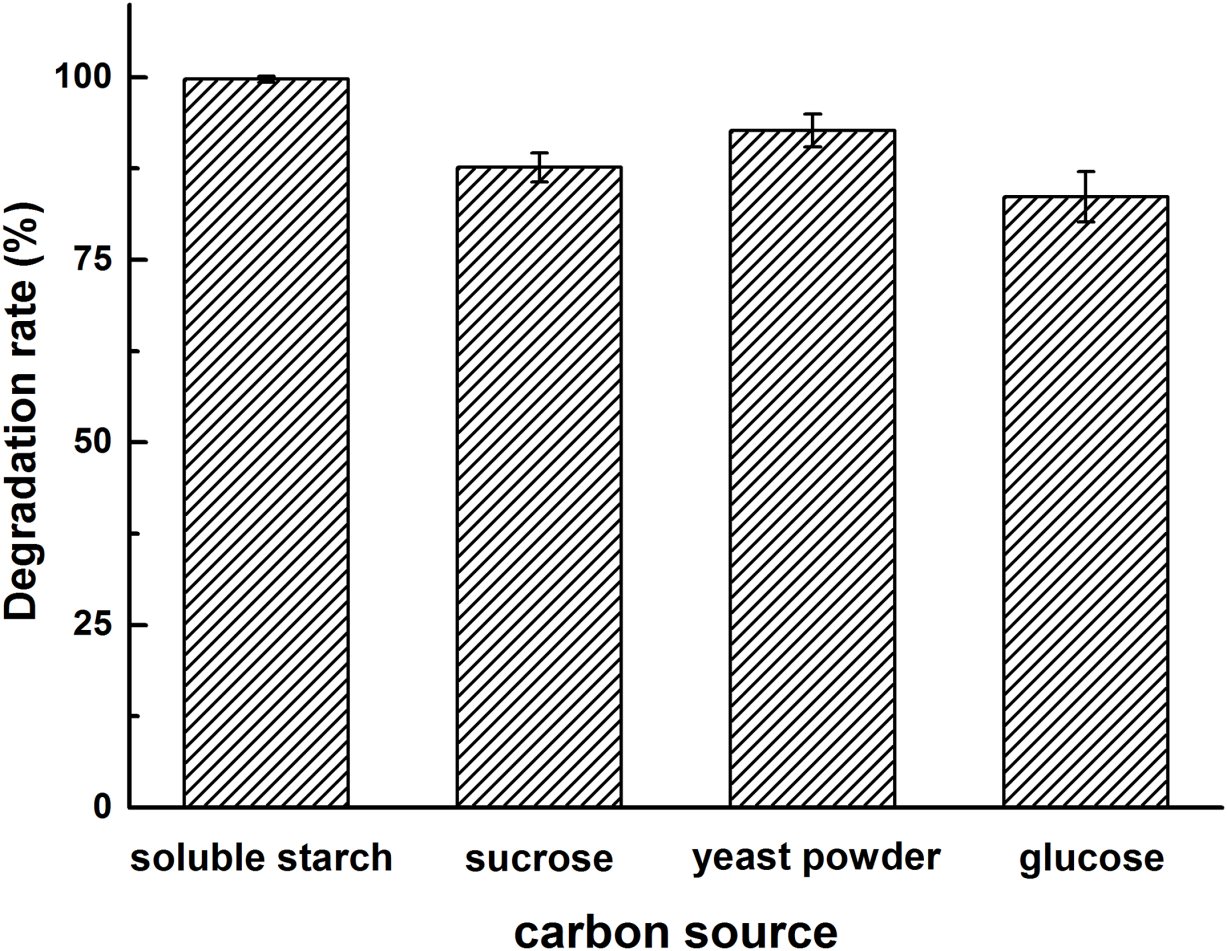
The effects of four different carbon sources on diuron degradation by strain DP8-1. Values were the mean of three replicates with standard deviation.

To evaluate carbon source effect on the degradation of diuron by strain DP8-1, soluble starch, sucrose, yeast extract, and glucose were added to MSM as supplementary carbon sources in this study. Durion as the sole carbon source in MSM was also used to compare the degradation efficiency. As shown in Fig 3, the degradation rate of diuron were 99.75%, 87.67%, 92.70%, and 83.65% in the presence of soluble starch, sucrose, yeast extract, and glucose, respectively, while lower degradation rate (61.02%) of diuron was observed in MSM without additional carbon sources. Abiotic degradation was negligible in non-inoculated control. The data in Fig 3 showed that four different carbon sources promoted diuron degradation after incubation for 3 days. Compared to other carbon sources studied, soluble starch was the best carbon source. Thus, soluble starch was chosen as assisted carbon source for the biodegradation of diuron. The results indicated that diuron degradation by strain DP8-1 may be undergo a co-metabolic process, which was a very universal phenomenon in the biodegradation of herbicides [37]. Additionally, the degradation rate of diuron obtained in MSM with biotin was 66.44%. The result showed that the growth and degradability of strain DP8-1 was slightly affected by biotin and strain DP8-1 could well grow without biotin. Thus, the degradation experiments were carried out without the addition of biotin.

### Effect of initial concentration on the degradation of diuron by strain DP8-1

**Fig 4:**
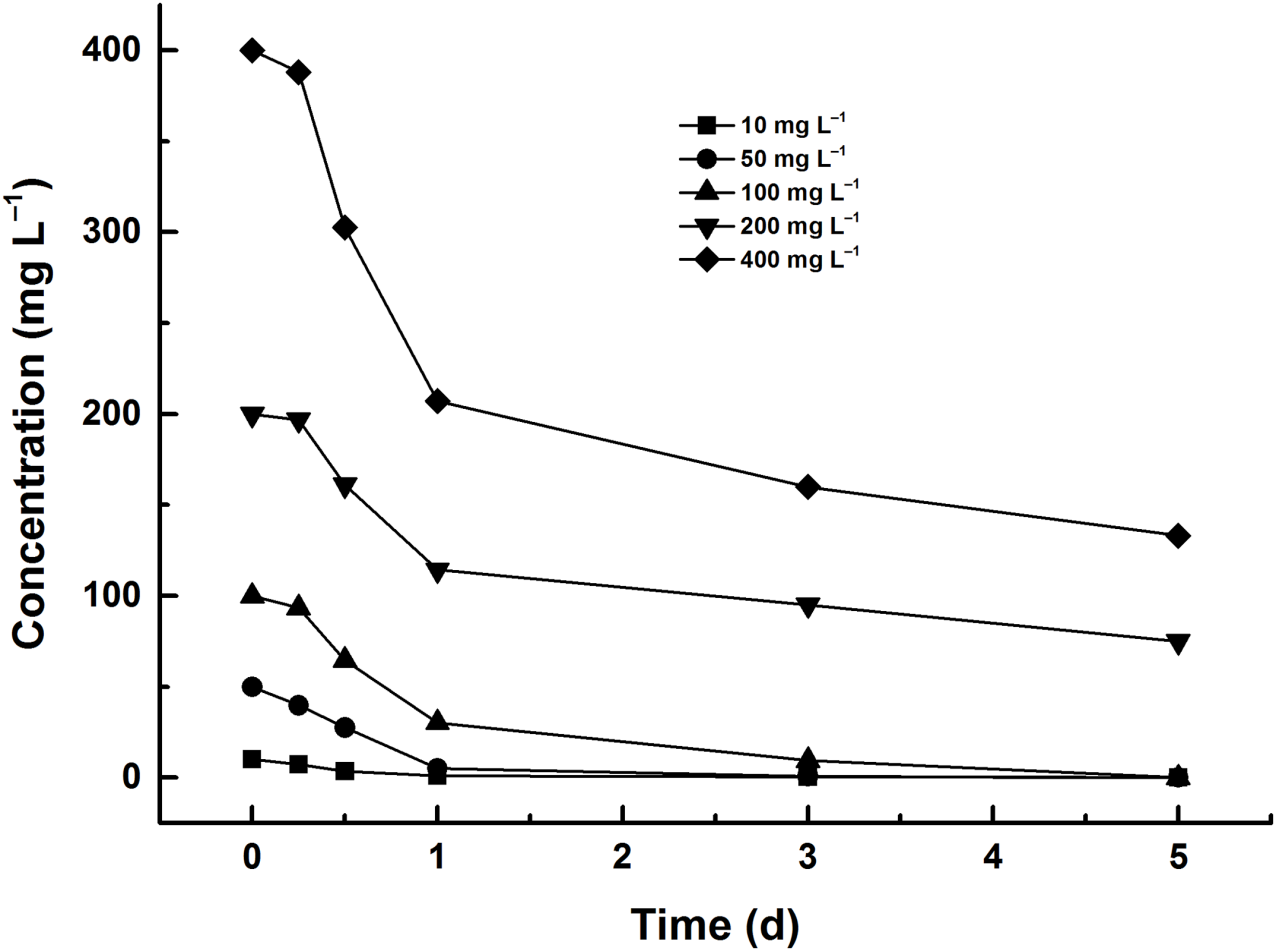
Degradation dynamics of diuron with different initial concentrations in MSM supplemented with 0.5% soluble starch as an additional carbon source by strain DP8-1.

To determine the effect of initial concentration of diuron on the degradation efficiency, the degradation experiments were conducted out with the concentration of diuron up to 400 mg L^‒1^ under the optimal conditions and the results were illustrated in Fig 4. Observed from Fig 4, the degradation rate were 95.70%, 98.42%, 90.47%, 52.47%, and 60.04% at the diuron concentrations of 10, 50 100, 200, and 400 mg L^‒1^ within 3 days, respectively. The degradation rate preliminarily increased with the increasing initial concentration and diuron could be effectively degraded when the concentration was below 100 mg L^‒1^. It may be due to that diuron could act as energy source and promote the growth of strain DP8-1, thereby resulted in the effective biodegradation. Additionally, it was obvious that the degradation efficiency of diuron by strain DP8-1 was weakened at higher diuron concentration. It was possible that the growth of strain DP8-1 or activity of degrading enzymes from strain DP8-1 had been inhibited with the further increase of diuron concentration. When diuron concentration was 50 mg L^‒1^, the largest degradation rate was obtained. Thus, 50 mg L^‒1^ was chosen as the initial concentration of diuron.

### Biodegradation of diuron by strain DP8-1

**Fig 5:**
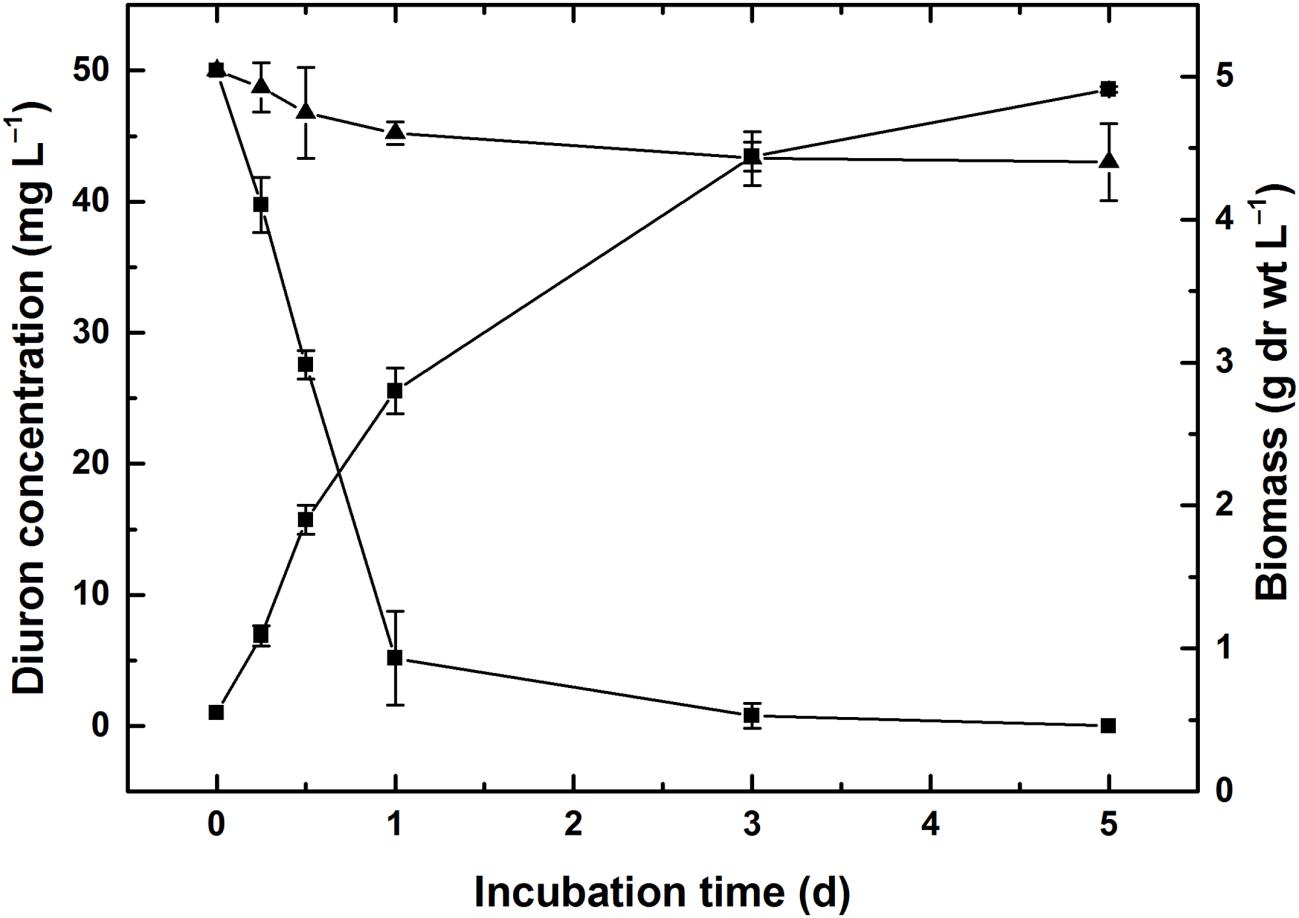
Biomass of strain DP8-1 (■), and degradation kinetics of diuron by strain DP8-1 (●) and *N*. *intermedia* BNCC144684 (▲) in MSM supplemented with 0.5% soluble starch as an additional carbon source.

The diuron degradation and strain growth patterns were measured under the optimal conditions and the results were illustrated in Fig 5. The concentration of diuron residue was rapidly decreasing in the first day, then slowly changed in the next two to three days, finally slight reduction at the last two days. The concentration change indicated that diuron was rapidly degraded by strain DP8-1 during the first day and more than 98% degradation was detected within 3 days. No significant change in diuron concentration was observed in non-inoculated culture. The degradation rate of diuron by *N*. *intermedia* BNCC144684 was 13.97% after incubation for 5 days. The comparisons showed that the degradation of diuron was due to strain DP8-1 and strain DP8-1 possessed prominent biodegradability to diuron. Meanwhile, the dry weight of mycelium constantly increased from 0.55 to 4.91 g during the process of diuron degradation. Diuron decrease associated with fungal biomass increase indicated that strain DP8-1 could utilize diuron for their growth.

### Biodegradation of various phenylurea herbicides by strain DP8-1

The ability of strain DP8-1 to degrade various phenylurea herbicides were shown in Table 4, which were tested with the phenylurea herbicides at 50 mg L^‒1^ after 7 days of incubation in amendment MSM. Interestingly, isoproturon was completely degraded by strain DP8-1. The degradation rates for fenuron, metobromuron, chlorbromuron, and linuron were 76.95%, 85.58%, 78.18%, and 89.63%, respectively. However, monuron was only degraded 40.26% at the end of experiment. The results showed that strain DP8-1 could degrade a broad spectrum of phenylurea herbicides. To our knowledge, it has not been reported that an endophytic fungus *N*. *intermedia* can be used for the degradation of phenylurea herbicides. These findings provided important information for applying *N*. *intermedia* in environmental protection and pharmaceutical industry.

**Table 4:** Degradation of various phenylurea herbicides by strain DP8-1 after 7 days of incubation in amendment MSM.

Phenylurea herbicides	Degradation rate (%)^a^
fenuron	76.95±5.91
monuron	40.26±7.34
metobromuron	85.58±4.19
isoproturon	100.00±0.00
chlorbromuron	78.18±3.37
linuron	89.63±1.95

^a^ Values are the mean of three replicates with standard deviation.

### Determination of the degradation products of diuron

The diuron metabolic products in culture medium were extracted and identified by LC-MS and HPLC. Based on LC-MS analysis, two major degradation products were detected with retention times of 5.893 and 7.697 representing metabolites A and B, while diuron had a retention time of 9.674 in positive ion mode (S2 Fig). Metabolite A in negative ion mode showed a molecular ion at m/z 202.70 [M+H]^‒^ and a characteristic fragment ion peak at m/z 160.10. Metabolite B showed molecular ions at m/z 218.80 and 216.80 [M+H]^+^, and characteristic fragment ion peaks at m/z 163.90 and 160.00 in positive and negative ion mode, respectively. The two products were preliminarily identified as DCPU and DCPMU, respectively. Two metabolites were also confirmed by the same retention time with their standard chemicals in HPLC analysis (S3 Fig). Their structural formula were showed in S4 Fig. According to the identified results, we speculated that strain DP8-1 degraded diuron through sequential N-dealkylations (S4 Fig).

**Fig 6:**
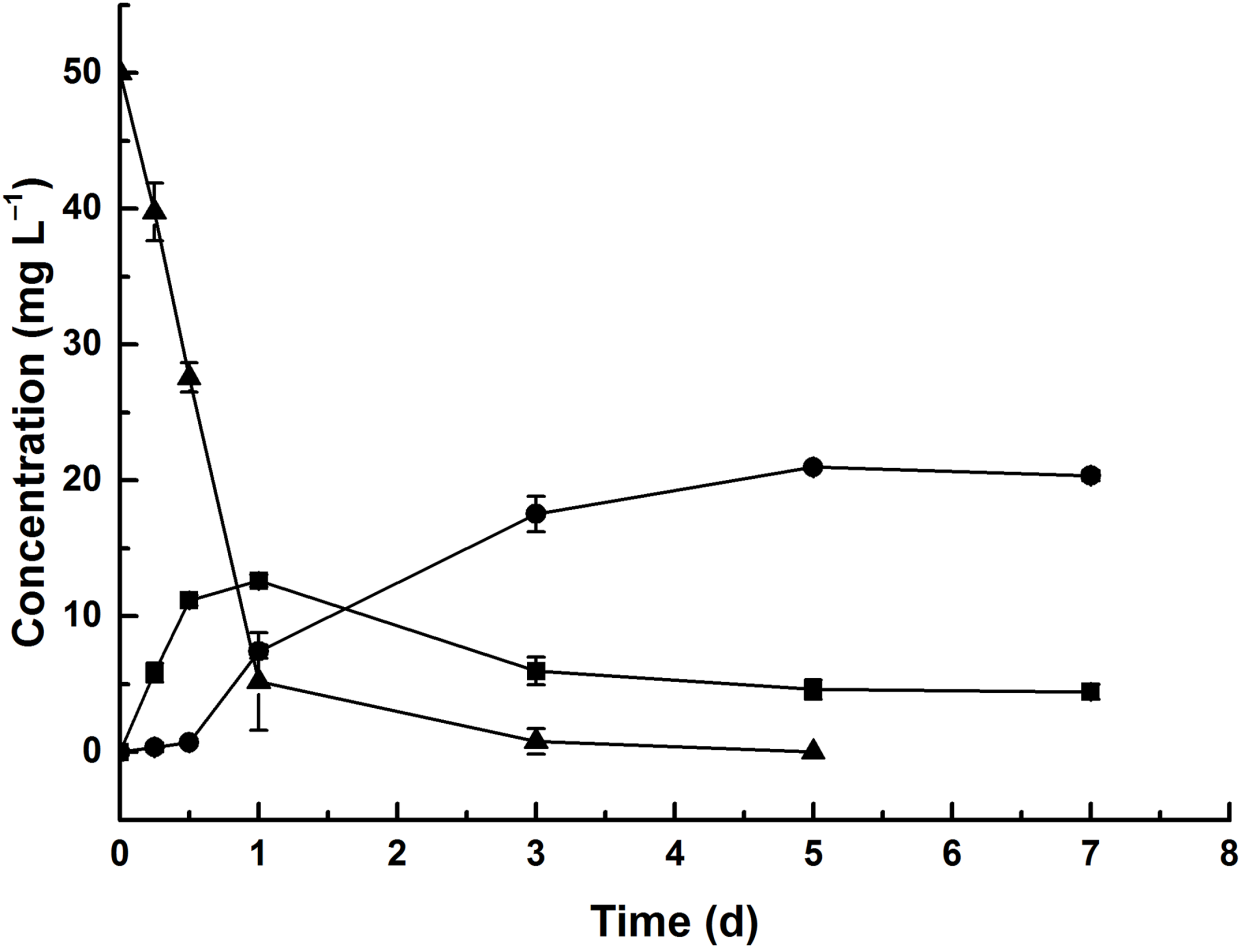
Diuron degradation (▲) and the formation of DCPMU (■), DCPU (●) during the degradation.

During the biodegradation process by strain DP8-1, the concentration of DCPMU reached 12.62 mg L^−1^ after cultivation for 1 day and decreased to 4.43 g mL^−1^ at the end of the experiment. The metabolite DCPU was detected with a maximal concentration of 20.97 mg L^−1^ after cultivation for 5 days (Fig 6). An unidentified peak with the retention time of 5.33 min was also observed during the whole cultivation. In previous studies, it has been reported that some fungal strains could degrade diuron to DCPMU and DCPU in liquid cultures [9, 19, 38, 39]. Some studies conducted in liquid cultures also showed that many fungal strains could finally degrade diuron to 3,4-DCA [9, 19, 38, 40]. However, no 3,4-DCA was detected in the whole degradation process by strain DP8-1. It may be due to that strain DP8-1 rapidly degraded 3,4-DCA to other metabolites. Indeed, we found that strain DP8-1 could utilize 3,4-DCA as the sole carbon and energy source in MSM (data not shown). These results indicated that the endophytic fungus DP8-1 may appear new metabolic pathway and mechanism for the degradation and metabolism of diuron. In future, degradation pathway would be confirmed by ^14^C-labelled method, and metabolites would be identified by nuclear magnetic resonance.

### Soil bioremediation experiment

**Fig 7:**
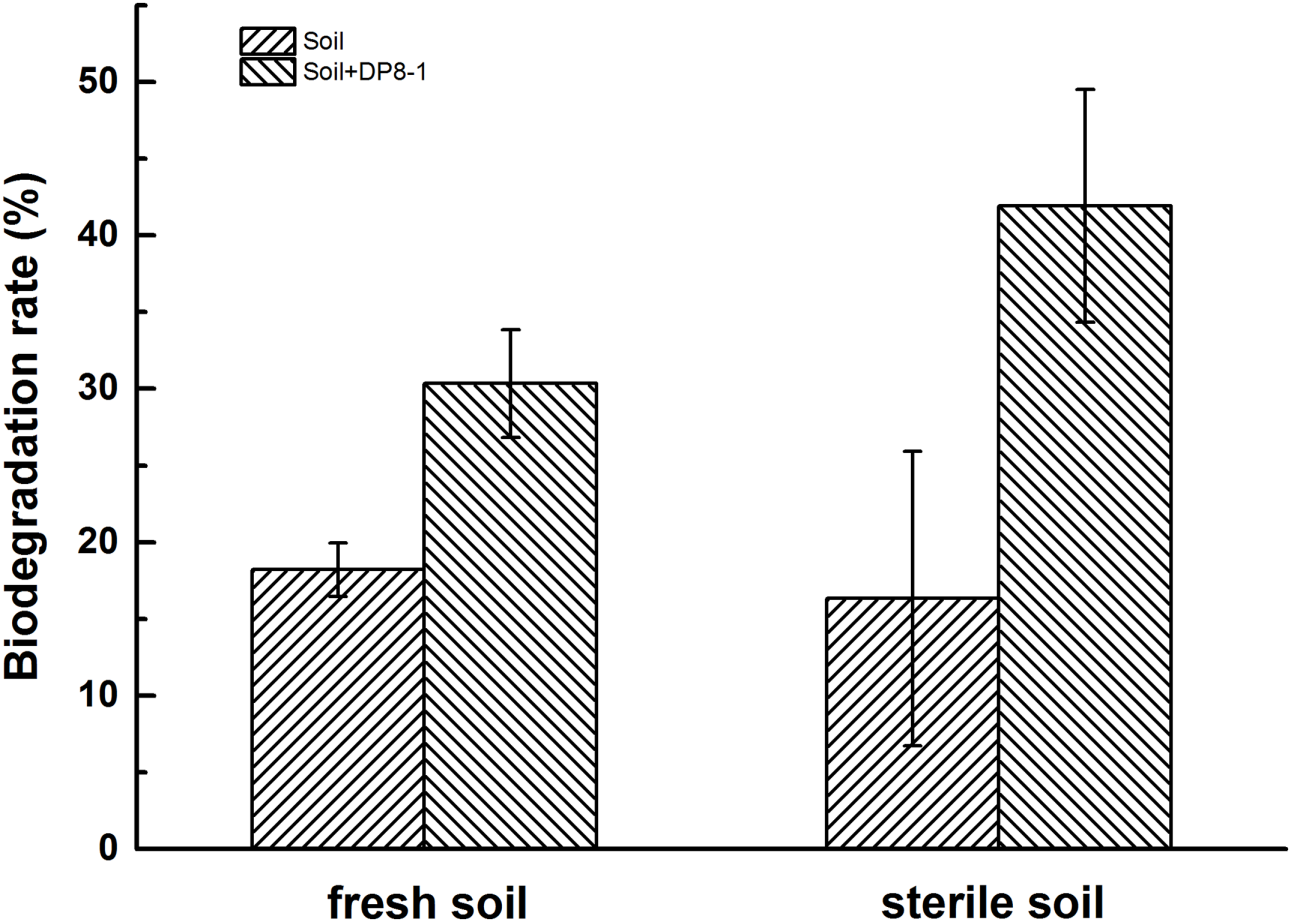
Degradation of diuron in different soil treatments after 20 days of incubation. Error bars represented the standard deviation of the mean.

Degradation of diuron in different soil treatments were measured after 20 days of incubation and the result was shown in Fig 7. In fresh soil, the degradation rates of diuron were 18.21% and 30.33% for inoculating without and with strain DP8-1, respectively. In contrast, diuron in sterile soil was removed up to 16.34% and 41.92% during the same time period. There existed significant difference between all treatments by LSD analysis (p = 0.05). Diuron was slightly degraded without strain DP8-1, which may be due to chemical hydrolysis. However, the degradation rate of diuron was increased in the present of strain DP8-1. The results demonstrated that strain DP8-1 significantly enhanced the degradation of diuron in soil samples.

The residues of diuron in sterile soil, fresh soil, and strain DP8-1 amended soils were also determined at 10, 40, and 60 days. The corresponding first-order kinetic rate coefficients and half-lives were shown in Table 5. Degradation efficiency in fresh soil was much better than that in sterile soil, demonstrating that soil microbes played an important role in diuron degradation [41]. After inoculated with strain DP8-1, diuron degradation was 2.28 and 1.83 times faster than in fresh and sterile soils. The half-lives illustrated in this experiment were in accordance with several previous reports with half-lives ranging from several weeks to several months [2, 42, 43, 44]. The diversity of diuron half-life in soil was due to that it was affected by many factors such as soil type, soil constitution, soil microbiota, tested strain, initial concentration of diuron, and experimental condition, etc. This experiment showed that it was worthy of further investigation to develop strain DP8-1 as a bioremediation agent for phenylurea herbicide contaminated environments.

**Table 5:** Degradation dynamics of diuron by strain DP8-1 in different soils.

Soil treatment	Equation	Correlation coefficient (R^2^)	Half-life (day)
Fresh soil	y = 81.245e^−0.051x^	R^2^ = 0.7931	13.59
Fresh soil+DP8-1	y = 135.66e^−0.116x^	R^2^ = 0.8930	5.97
Sterile soil	y = 57.011e^−0.018x^	R^2^ = 0.8919	38.50
Sterile soil+DP8-1	y = 54.782e^−0.033x^	R^2^ = 0.9920	21.00

## Conclusions

An endophytic fungus strain DP8-1, isolated from the root of sugarcane, could degrade diuron in liquid medium and soil. The strain exhibited a broad degradation spectrum to phenylurea herbicides including fenuron, monuron, metobromuron, isoproturon, chlorbromuron, and linuron. The main degradative pathway of diuron by this strain was sequential N-dealkylations. The feasibility of using the isolated strain *N*. *intermedia* DP8-1 to remove and detoxify the diuron contamination in practice needs to be investigated in detail in future.

## Supporting information

S1 Fig Morphological characteristics of the fungal strain DP8-1. (a) Colony morphology on PD agar plate after incubation for 7 days. Fungal hypha (b) and conidia (c) observed under a light microscope.
(TIF)

S2 Fig Mass spectra of metabolic products (a) DCPU, (b) DCPMU, and (c) diuron. (TIF)

S3 Fig HPLC spectra of (a) diuron (12.426 min), (b) DCPU (6.342 min), DCPMU (9.088 min), 3,4-DCCA (11.511 min), and 3,4-DCA (16.311 min), and (c) metabolites after 3 days of inoculation with strain DP8-1. (TIF)

S4 Fig Degradation pathway of diuron by strain DP8-1. DCPMU: [1-(3,4-dichlorophenyl)-3-methylurea], DCPU: [1-(3,4-dichlorophenyl) urea].
(TIF)

## References

[1] Coelho-Moreira JdS, Bracht A, de Souza ACdS, Oliveira RF, de Sa-Nakanishi AB, de Souza CGM, et al Degradation of diuron by Phanerochaete chrysosporium: role of ligninolytic enzymes and cytochrome P450. BioMed research international. 2013;2013:1–9. doi: 10.1155/2013/251354 24490150 10.1155/2013/251354 PMC3892757

[2] Fontecha-Cámara MA, López-Ramón MV, Pastrana-Martínez LM, Moreno-Castilla C. Kinetics of diuron and amitrole adsorption from aqueous solution on activated carbons. Journal of Hazardous Materials. 2008;156(1–3):472–7. doi: 10.1016/j.jhazmat.2007.12.043 18241982 10.1016/j.jhazmat.2007.12.043

[3] Louchart X, Voltz M, Andrieux P, Moussa R. Herbicide transport to surface waters at field and watershed scales in a Mediterranean vineyard area. Journal of Environmental Quality. 2001;30(3):982–91. doi: 10.2134/jeq2001.303982x 11401289 10.2134/jeq2001.303982x

[4] Thurman EM, Bastian KC, Mollhagen T. Occurrence of cotton herbicides and insecticides in playa lakes of the High Plains of West Texas. Science of The Total Environment. 2000;248(2–3):189–200. doi: 10.1016/S0048-9697(99)00542-2 10805239 10.1016/s0048-9697(99)00542-2

[5] Inoue MH, Possamai ACS, Mendes KF, Ben R, de Matos AKA, dos Santos EG. Potential of leaching of herbicide used in sugar cane in soils contrasting. Bioscience Journal. 2014;30(15):659–65.

[6] Langeron J, Sayen S, Couderchet M, Guillon E. Leaching potential of phenylurea herbicides in a calcareous soil: comparison of column elution and batch studies. Environmental Science and Pollution Research. 2014;21(7):4906–13. doi: 10.1007/s11356-012-1244-y 23097070 10.1007/s11356-012-1244-y

[7] Huovinen M, Loikkanen J, Naarala J, Vahakangas K. Toxicity of diuron in human cancer cells. Toxicology in Vitro. 2015;29(7):1577–86. doi: 10.1016/j.tiv.2015.06.013 26086120 10.1016/j.tiv.2015.06.013

[8] Canna-Michaelidou S, Nicolaou A-S. Evaluation of the genotoxicity potential (by Mutatox™ test) of ten pesticides found as water pollutants in Cyprus. Science of the Total Environment. 1996;193(1):27–35. doi: 10.1016/S0048-9697(96)05322-3 9111768 10.1016/s0048-9697(96)05322-3

[9] Sorensen SR, Bending GD, Jacobsen CS, Walker A, Aamand J. Microbial degradation of isoproturon and related phenylurea herbicides in and below agricultural fields. Fems Microbiology Ecology. 2003;45(1):1–11. doi: 10.1016/S0168-6496(03)00127-2 19719601 10.1016/S0168-6496(03)00127-2

[10] Tixier C, Sancelme M, Ait-Aissa S, Widehem P, Bonnemoy F, Cuer A, et al Biotransformation of phenylurea herbicides by a soil bacterial strain, *Arthrobacter* sp N2: structure, ecotoxicity and fate of diuron metabolite with soil fungi. Chemosphere. 2002;46(4):519–26. doi: 10.1016/s0045-6535(01)00193-x 11838430 10.1016/s0045-6535(01)00193-x

[11] Widehem P, Ait-Aissa S, Tixier C, Sancelme M, Veschambre H, Truffaut N. Isolation, characterization and diuron transformation capacities of a bacterial strain Arthrobacter sp N2. Chemosphere. 2002;46(4):527–34. doi: 10.1016/s0045-6535(01)00192-8 11838431 10.1016/s0045-6535(01)00192-8

[12] Dellamatrice PM, Monteiro RTR. Isolation of diuron-degrading bacteria from treated soil. Brazilian Archives of Biology and Technology. 2004;47(6):999–1003. doi: 10.1590/s1516-89132004000600020

[13] Castillo MA, Felis N, Aragon P, Cuesta G, Sabater C. Biodegradation of the herbicide diuron by *streptomycetes* isolated from soil. International Biodeterioration & Biodegradation. 2006;58(3–4):196–202. doi: 10.1016/j.ibiod.2006.06.020

[14] Sharma P, Suri CR. Biotransformation and biomonitoring of phenylurea herbicide diuron. Bioresource Technology. 2011;102(3):3119–25. doi: 10.1016/j.biortech.2010.10.076 21075624 10.1016/j.biortech.2010.10.076

[15] Sharma P, Chopra A, Cameotra SS, Suri CR. Efficient biotransformation of herbicide diuron by bacterial strain *Micrococcus* sp. PS-1. Biodegradation. 2010;21(6):979–87. doi: 10.1007/s10532-010-9357-9 20401520 10.1007/s10532-010-9357-9

[16] Ngigi A, Getenga Z, Boga H, Ndalut P. Biodegradation of phenylurea herbicide diuron by microorganisms from long-term-treated sugarcane-cultivated soils in Kenya. Toxicological and Environmental Chemistry. 2011;93(8):1623–35. doi: 10.1080/02772248.2011.595718

[17] Batisson I, Pesce S, Besse-Hoggan P, Sancelme M, Bohatier J. Isolation and characterization of diuron-degrading bacteria from lotic surface water. Microbial Ecology. 2007;54(4):761–70. doi: 10.1007/s00248-007-9241-2 17450392 10.1007/s00248-007-9241-2

[18] Ellegaard-Jensen L, Aamand J, Kragelund BB, Johnsen AH, Rosendahl S. Strains of the soil fungus *Mortierella* show different degradation potentials for the phenylurea herbicide diuron. Biodegradation. 2013;24(6):765–74. doi: 10.1007/s10532-013-9624-7 23361127 10.1007/s10532-013-9624-7

[19] Sorensen SR, Juhler RK, Aamand J. Degradation and mineralisation of diuron by *Sphingomonas* sp. SRS2 and its potential for remediating at a realistic μg L^‒1^ diuron concentration. Pest Management Science. 2013;69(11):1239–44. doi: 10.1002/ps.3490 23494959 10.1002/ps.3490

[20] Khan Z, Roman D, Kintz T, Alas MD, Yap R, Doty S. Degradation, phytoprotection and phytoremediation of phenanthrene by endophyte *Pseudomonas putida*, PD1. Environmental Science & Technology. 2014;48(20):12221–8. doi: 10.1021/es503880t 25275224 10.1021/es503880t PMC12476886

[21] Ozawa T, Yoshida R, Wakashiro Y, Hase H. Improvement of simazine degradation by inoculation of corn and soybean plants with rhizobacteria. Soil Science and Plant Nutrition. 2004;50(8):1295–9.

[22] Kuklinsky-Sobral HL, Araujo WL, Mendes R, Pizzirani-Kleiner AA, Azevedo JL. Isolation and characterization of endophytic bacteria from soybean *(Glycine max)* grown in soil treated with glyphosate herbicide. Plant and Soil. 2005;273(1–2):91–9. doi: 10.1007/s11104-004-6894-1

[23] Liu M, Luo K, Wang Y, Zeng A, Zhou X, Luo F, et al Isolation, identification and characteristics of an endophytic quinclorac degrading bacterium *Bacillus megaterium* Q3. Plos One. 2014;9(9). doi: 10.1371/journal.pone.0108012 25244184 10.1371/journal.pone.0108012 PMC4171507

[24] Glass NL, Donaldson GC. Development of primer sets designed for use with the PCR to amplify conserved genes from filamentous ascomycetes. Applied and Environmental Microbiology. 1995;61(4):1323–30. 7747954 10.1128/aem.61.4.1323-1330.1995 PMC167388

[25] Johannesson HS, Johannesson KHP, Stenlid J. Development of primer sets to amplify fragments of conserved genes for use in population studies of the fungus *Daldinia loculata*. Molecular Ecology. 2000;9(3):375–7. doi: 10.1046/j.1365-294x.2000.00874-6.x 10736039 10.1046/j.1365-294x.2000.00874-6.x

[26] Bunyard BA, Nicholson MS, Royse DJ. A systematic assessment of *Morchella* using RFLP analysis of the 28S ribosomal RNA gene. Mycologia. 1994;86(6):762–72.

[27] Vilgalys R, Hester M. Rapid genetic identification and mapping of enzymatically amplified ribosomal DNA from several *Cryptococcus* species. Journal of Bacteriology. 1990;172(8):4238–46. 2376561 10.1128/jb.172.8.4238-4246.1990 PMC213247

[28] Nygren K, Strandberg R, Wallberg A, Nabholz B, Gustafsson T, García D, et al A comprehensive phylogeny of *Neurospora* reveals a link between reproductive mode and molecular evolution in fungi. Molecular Phylogenetics and Evolution. 2011;59(3):649–66. doi: 10.1016/j.ympev.2011.03.023 21439389 10.1016/j.ympev.2011.03.023

[29] Guo W-Q, Ren N-Q, Wang X-J, Xiang W-S, Ding J, You Y, et al Optimization of culture conditions for hydrogen production by *Ethanoligenens harbinense* B49 using response surface methodology. Bioresource Technology. 2009;100(3):1192–6. doi: 10.1016/j.biortech.2008.07.070 18793840 10.1016/j.biortech.2008.07.070

[30] Shareef A, Page D, Vanderzalm J, Williams M, Gupta V, Dillon P, et al Biodegradation of simazine and diuron herbicides under aerobic and anoxic conditions relevant to managed aquifer recharge of storm water. Clean-Soil Air Water. 2014;42(6):745–52. doi: 10.1002/clen.201300092

[31] Zhou L, Zheng W, Ji YF, Zhang JF, Zeng C, Zhang Y, et al Ferrous-activated persulfate oxidation of arsenic(III) and diuron in aquatic system. Journal of Hazardous Materials. 2013;263:422–30. doi: 10.1016/j.jhazmat.2013.09.056 24220194 10.1016/j.jhazmat.2013.09.056

[32] Zhang X-H, Zhang G-S, Zhang Z-H, Xu J-H, Li S-P. Isolation and characterization of a dichlorvos-degrading strain DDV-1 of *Ochrobactrum* sp. Pedosphere. 2006;16(1):64–71. doi: 10.1016/S1002-0160(06)60027-1

[33] Perkins DD. Neurospora: the organism behind the molecular revolution. Genetics. 1992;130(4):687–701. 1582553 10.1093/genetics/130.4.687 PMC1204921

[34] Song J, Gu J, Zhai Y, Wu W, Wang H, Ruan Z, et al Biodegradation of nicosulfuron by a *Talaromyces flavus* LZM1. Bioresource Technology. 2013;140:243–8. doi: 10.1016/j.biortech.2013.02.086 23707911 10.1016/j.biortech.2013.02.086

[35] Hou N, Feng F, Shi Y, Cao H, Li C, Cao Z, et al Characterization of the extracellular biodemulsifiers secreted by *Bacillus cereus* LH-6 and the enhancement of demulsifying efficiency by optimizing the cultivation conditions. Environmental Science and Pollution Research. 2014;21(17):10386–98. doi: 10.1007/s11356-014-2931-7 24777330 10.1007/s11356-014-2931-7

[36] Schenone AV, Conte LO, Botta MA, Alfano OM. Modeling and optimization of photo-Fenton degradation of 2,4-D using ferrioxalate complex and response surface methodology (RSM). Journal of Environmental Management. 2015;155:177–83. doi: 10.1016/j.jenvman.2015.03.028 25819569 10.1016/j.jenvman.2015.03.028

[37] Luo W, Zhao Y, Ding H, Lin X, Zheng H. Co-metabolic degradation of bensulfuron-methyl in laboratory conditions. Journal of Hazardous Materials. 2008;158(1):208–14. doi: 10.1016/j.jhazmat.2008.02.115 18417283 10.1016/j.jhazmat.2008.02.115

[38] Castanon-Gonzalez JH, Galindez-Mayer J, Ruiz-Ordaz N, Rocha-Martinez L, Pena-Partida JC, Marron-Montiel E, et al Biodegradation of the herbicide Diuron in a packed bed channel and a double biobarrier with distribution of oxygenated liquid by airlift devices: influence of oxygen limitation. New Biotechnology. 2016;33(1):7–15. doi: 10.1016/j.nbt.2015.07.002 26241887 10.1016/j.nbt.2015.07.002

[39] Ellegaard-Jensen L, Knudsen BE, Johansen A, Albers CN, Aamand J, Rosendahl S. Fungal-bacterial consortia increase diuron degradation in water-unsaturated systems. Science of the Total Environment. 2014;466:699–705. doi: 10.1016/j.scitotenv.2013.07.095 23973535 10.1016/j.scitotenv.2013.07.095

[40] Batori V, Ferreira JA, Taherzadeh MJ, Lennartsson PR. Ethanol and protein from ethanol plant by-products using edible fungi *Neurospora intermedia* and *Aspergillus oryzae*. Biomed Research International. 2015 doi: 10.1155/2015/176371 26682213 10.1155/2015/176371 PMC4670849

[41] Yun LY, Xiao W, Yong ML, Ji FY, Jing QY, De FF. Fungal degradation of metsulfuron-methyl in pure cultures and soil. Chemosphere. 2005;60(4):460–6. doi: 10.1016/j.chemosphere.2005.01.015 15950038 10.1016/j.chemosphere.2005.01.015

[42] Devers-Lamrani M, Pesce S, Rouard N, Martin-Laurent F. Evidence for cooperative mineralization of diuron by *Arthrobacter* sp. BS2 and *Achromobacter* sp. SP1 isolated from a mixed culture enriched from diuron exposed environments. Chemosphere. 2014;117:208–15. doi: 10.1016/j.chemosphere.2014.06.080 25061887 10.1016/j.chemosphere.2014.06.080

[43] Rouchaud J, Neus O, Bulcke R, Cools K, Eelen H, Dekkers T. Soil dissipation of diuron, chlorotoluron, simazine, propyzamide, and diflufenican herbicides after repeated applications in fruit tree orchards. Archivesof Environmental Contamination and Toxicology. 2000;39(1):60–5. doi: 10.1007/s002440010080 10790503 10.1007/s002440010080

[44] El Sebaï T, Devers M, Lagacherie B, Rouard N, Soulas G, Martin-Laurent F. Diuron mineralisation in a Mediterranean vineyard soil: impact of moisture content and temperature. Pest Management Science. 2010;66(9):988–95. doi: 10.1002/ps.1971 20730991 10.1002/ps.1971

