# Supplementary figures and images for: Biodegradation of diuron by an endophytic fungus Neurospora intermedia DP8-1 isolated from sugarcane and its potential for remediating diuron-contaminated soils
Source: PLoS One. 2017 Aug 15;12(8):e0182556. doi: 10.1371/journal.pone.0182556 (PMC5557362; doi:10.1371/journal.pone.0182556)

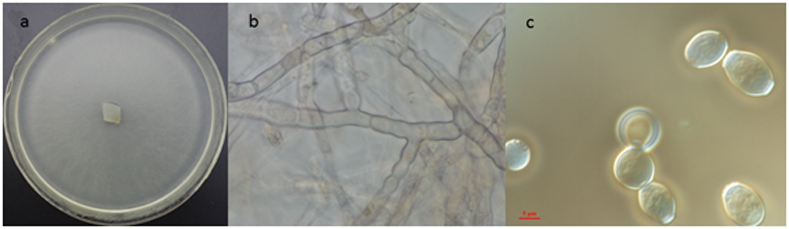

Supplement: S1 Fig — (a) Colony morphology on PD agar plate after incubation for 7 days. Fungal hypha (b) and conidia (c) observed under a light microscope. (TIF) [file pone.0182556.s001.tif]

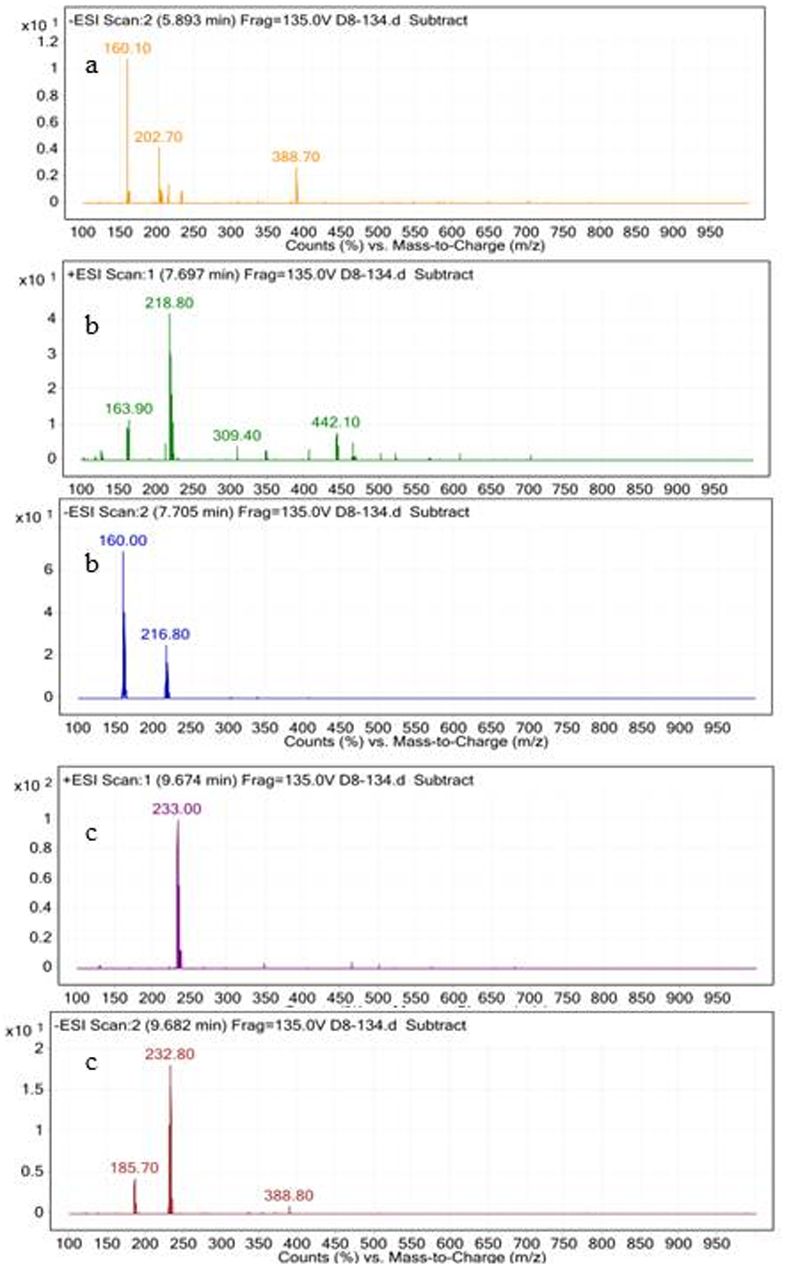

Supplement: S2 Fig — (TIF) [file pone.0182556.s002.tif]

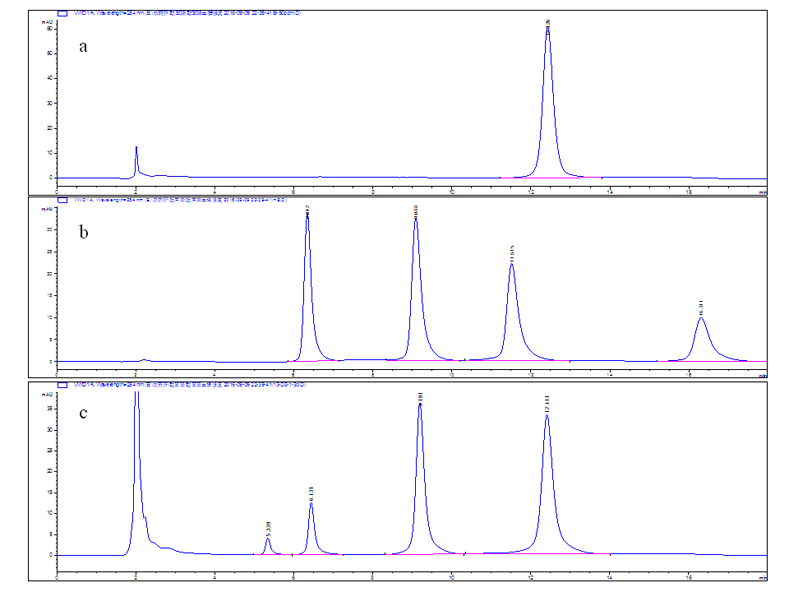

Supplement: S3 Fig — (TIF) [file pone.0182556.s003.tif]

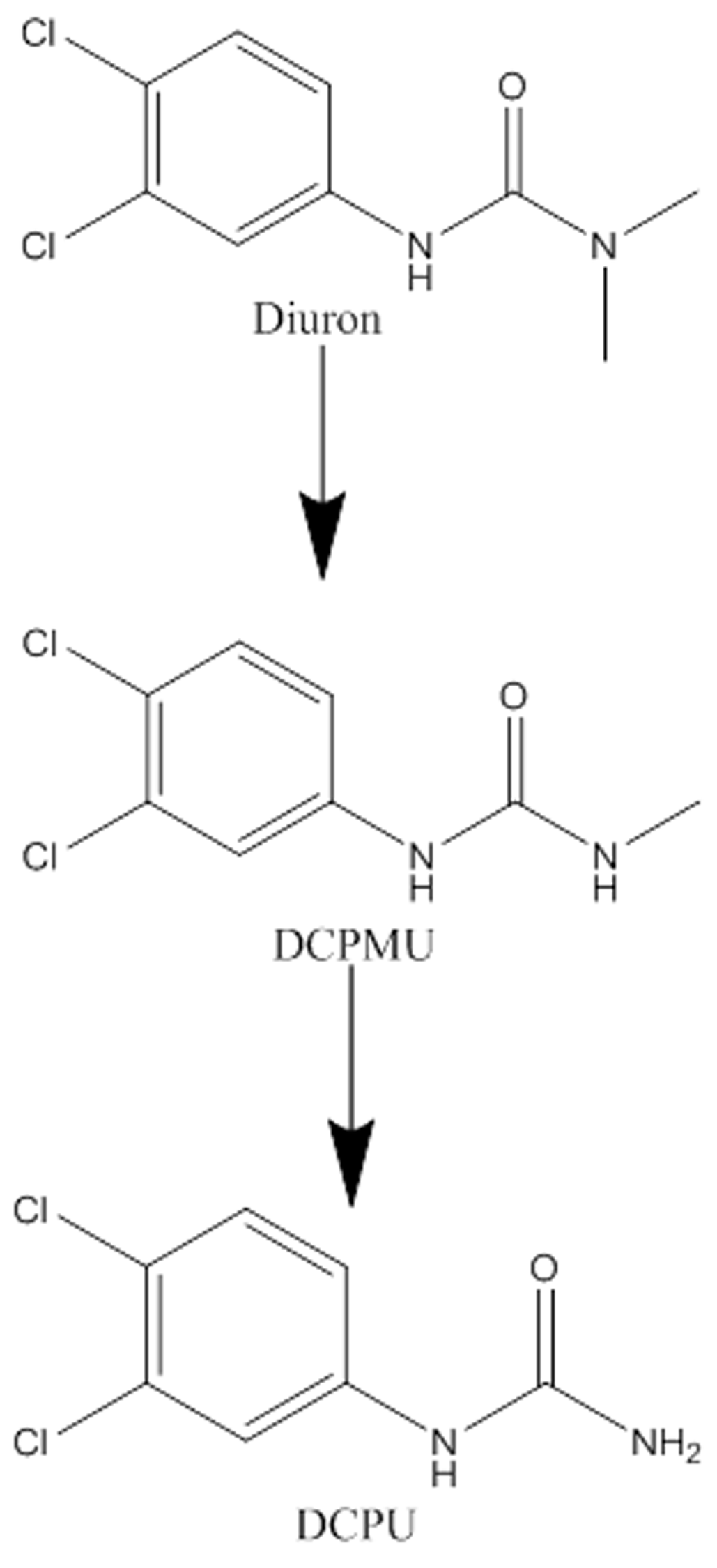

Supplement: S4 Fig — DCPMU: [1-(3,4-dichlorophenyl)-3-methylurea], DCPU: [1-(3,4-dichlorophenyl) urea]. (TIF) [file pone.0182556.s004.tif]
